# Difference in symptom severity between early and late grass pollen season in patients with seasonal allergic rhinitis

**DOI:** 10.1186/2045-7022-1-18

**Published:** 2011-12-21

**Authors:** Letty A de Weger, Thijs Beerthuizen, Jeannette M Gast-Strookman, Dirk T van der Plas, Ingrid Terreehorst, Pieter S Hiemstra, Jacob K Sont

**Affiliations:** 1 Department of Pulmonology, Leiden University Medical Center, Leiden, The Netherlands; 2 Department of Medical Decision Making, Leiden University Medical Center, Leiden, The Netherlands; 3 Department of ENT and Dept of Pediatrics, Academic Medical Center, Amsterdam, The Netherlands

**Keywords:** Allergic rhinitis, grass pollen, seasonal allergic rhinitis, symptoms, seasonal variation

## Abstract

**Background:**

For the development of forecasts for seasonal allergic rhinitis symptoms, it is essential to understand the relationship between grass pollen concentrations and the symptoms of grass pollen allergic patients.

**Objective:**

The aim of this study was to delineate this relationship between seasonal allergic rhinitis symptoms and grass pollen concentrations in the Netherlands.

**Methods:**

Grass pollen allergic patients (n = 80 [2007] - 84 [2008]) were enrolled into the study. They were asked to enter their seasonal allergic rhinitis symptoms (runny nose, sneezing, blocked nose, post nasal drip, and eye symptoms) daily on a scale from 0 to 3 to the study centre either by short message service (SMS) or by internet from May-July 2007 and April-July 2008. Daily pollen counts were used to define the early and the late grass pollen season as the period 'before and during' respectively 'after' the first grass pollen peak (more than 150 pollen/m^3^).

**Results:**

At similar grass pollen concentrations, the daily mean of the individual maximum symptom scores reported in the early season were higher as compared to that reported in the late season [differences of -0.41 (2007) and -0.30 (2008)]. This difference could not be explained by medication use by the patients nor by co-sensitization to birch.

**Conclusions:**

We conclude that seasonal allergic rhinitis symptoms at similar grass pollen concentrations are more severe in the early flowering season as compared to those in the late flowering season. This finding is not only relevant for development of forecasts for seasonal allergic rhinitis symptoms but also for understanding symptom development and planning and analysis of clinical studies.

## Background

Allergic rhinitis is amongst the most common chronic diseases of the world. It results from IgE-mediated inflammation after allergen exposure of the nasal mucosa and includes symptoms such as rhinorrhea, nasal obstruction, nasal itching and sneezing [1]. The majority of these patients (70-80%) also report ocular symptoms [2].

Grass pollen exposure is one of the major causes of allergic rhinitis in many parts of the world [3,4] The grass family comprises more than 10,000 wind pollinated species world wide. Since these species flower in succession, grass pollen is present in the air during a relatively long period of approximately 3 months in Western Europe. It has been shown that among at least 12 of these species the IgE-binding allergens involved are very similar [5]. Since grass pollen allergic patients respond to these different grass pollen species, they may suffer from seasonal allergic rhinitis symptoms during a relatively long period.

In the Netherlands, a daily symptom forecast for grass pollen allergic patients is broadcasted by radio since 1980 [6] and is also published on Dutch Teletext and the internet. Forecasts for seasonal allergic rhinitis symptoms can help patients to timely adjust medication and daily activities in order to reduce symptoms. For accurate symptom forecasts it is relevant to understand the relationship between grass pollen concentrations and the symptoms of grass pollen allergic rhinitis during the season. However, studies on the development of seasonal allergic rhinitis symptoms in the course of the pollen season are scarce [7,8]. Despite the fact that symptoms are recorded during the pollen season in many clinical studies, and data from placebo treatment could be used to study the symptom severity during the season [9,10], so far this has not been reported. Furthermore, it should be noted that in most of these intervention studies a selected patient population is studied with more severe seasonal allergic rhinitis.

The aim of the present study was to delineate the relationship between grass pollen concentrations and symptom severity in Dutch grass pollen allergic patients during the season. Since the present study is part of a larger project that aims to develop a multi-day forecast for seasonal allergic rhinitis symptoms that is not selectively aimed at more severe patients, we recruited the patients for this study by advertisements in the media (news papers and radio) from the general population.

## Methods

### Study population

Patients aged between 18 - 60 years were recruited through advertisement in local media. Since pollen counts can differ in different regions of the Netherlands we included only patients living in a region approximately 25 km around the city of Leiden. All participants were subjected to: (i) screening of their medical history (general and allergy); (ii) performance of skin prick tests with pollen (grass, elder, birch, mugwort), house dust mite *(Dermatophagoides pteronyssinus *and *D. farinae*), dog and cat (extracts were a kind gift of HAL Allergy B.V); and (iii) a general physical examination and a nasal examination to exclude the existence of nasal pathology interfering with the scoring of seasonal allergic rhinitis symptoms. Patients with seasonal allergic rhinitis symptoms during the grass pollen season and a positive skin test to grass pollen, with or without mild asthma (defined as treatment with bronchodilating β2-agonists only) were included. Since only a minority of the patients have mono grass pollen allergy we also enrolled patients with a poly-sensitization in order to ensure external validity. Participants were permitted oral antihistaminics, eye drops (antihistaminic, cromoglicate) and nasal sprays (antihistaminic, cromoglicate, corticosteroid) for rhinoconjunctivitis treatment. Exclusion criteria were: (i) a clinically relevant pet allergy and the very pet at home; (ii) immunotherapy current and in the past; (iii) pregnancy or breast feeding; (iv) daily use of inhaled corticosteroids for asthma; (v) daily use of oral corticosteroids; (vi) other significant co morbidity (e.g. severe cardiovascular or pulmonary disease, malignancy, autoimmune diseases). In 2007, 80 patients were enrolled in the study. In 2008, 9 patients withdrew from this group due to migration or pregnancy and 13 new patients were enrolled. The study protocol was approved by the Medical Ethical Committee of the Leiden University Medical Center (LUMC) and patients signed an informed consent before enrolment.

### Symptom scores

Symptoms scores were collected from May 15 until July 31 in 2007 (11 weeks) and from April 16 until July 31 in 2008 (15 weeks). The late start in symptom score collection in 2007 was due to the fact that study approval by the Medical Ethical Committee lagged behind schedule. Patients were asked to submit daily symptom scores to the study centre either by internet or by short message services (SMS) between 5.00 PM and 8.30 AM. This tight time frame was chosen in order to reduce recall bias. They were asked to rate the severity of 5 seasonal allergic rhinitis symptoms (runny nose, sneezing, blocked nose, postnasal drip, and eye symptoms) on a four-point scale ranging from zero (no symptoms) to three (severe symptoms). Also the daily medication use was scored: number of oral antihistaminics, number of eye drops, nasal spray, and inhalation medication. Finally, patients reported whether they spent the day in the region. The participants were reminded every afternoon at 5.00 PM by a SMS from the study centre to send in the scores.

### Pollen counts

Pollen was collected by a Hirst type volumetric spore-trap (Burkard Manufacturing Co. Limited, Hertfordshire, England) located on the roof of the 6^th ^floor (approx 20 m above street level) of the LUMC [6]. The sampler was operated weekly and the microscopic slides were scanned in three longitudinal bands covering a total area corresponding to 1 m^3 ^of sampled air in 24 h. The resulting counts are shown as average of daily pollen grains per m^3 ^of air.

### Statistical analysis

Compliance was calculated as the proportion of actual entries in the database to the number of expected entries per patient. Scores from outside the region were discarded in the analysis. From the categorized symptom scores daily means of the each one of the five symptom scores were calculated (e.g. mean score for sneezing). The daily mean medication use was calculated as the average of the total medication use per patient per day. Since there is substantial between-patient variation in symptoms having the greatest clinical impact and our interest was merely in prediction of between-day variations, we took the maximum score of either one of the symptom scores per patient per day as individual measure of symptom severity. The maximum score per day for either one of the symptoms can be considered as a measure for the most relevant symptom for a particular patient. Subsequently, we calculated the mean maximum symptom score as a daily measure of severity of seasonal allergic rhinitis for further analyses. All statistical analyses were performed with the statistical software package Intercooled STATA 11.0 (StataCorp, TX, USA).

The grass pollen season in 2007 ran from May 24, when 1% of annual sum was exceeded, to August 3, when 95% of total sum was reached. In 2008 the grass pollen season ran from May 21 to July 30. The period was defined as the early season, i.e. the period before and during the first very high grass pollen concentrations (more than 150 pollen/m^3^), being before June 12^th ^and May 31^st ^in 2007 and 2008, respectively. The late season was defined as the period after the first grass pollen peak. The relationship between the mean maximum symptom score (dependent) and the grass pollen concentration (independent) and the period (covariate) was analysed by multiple linear regression analysis.

In addition we assessed whether a potential period effect (i.e. late and early season) on the relationship between seasonal allergic rhinitis symptoms and grass pollen concentration could be explained by medication use or other clinically relevant allergies. Therefore, we performed an ordered logistic regression analysis with the individual maximum symptom score as the dependent variable and one confounding factor at a time in the model, clustered by patient to account for repeated observations within individuals (STATA, module ologit). The following factors were included in the model: (i) the dichotomized daily medication use per patient of either one of the permitted medications; (ii) the dichotomized daily use of nasal steroid spray for those patients using nasal steroid medication to reduce their seasonal allergic rhinitis symptoms; (iii) sensitization for birch pollen and house dust mite. Furthermore, in order to assess whether a potential period effect was influenced by allergic sensitization to house dust mite or birch pollen, we included the interaction term between period and the dichotomized allergic sensitization by skin prick tests for birch pollen (Birch^+^) or the clinically relevant allergy for house dust mite (HDM^+^) in the model. For the analysis with birch pollen sensitized patients a dichotomised parameter for the birch pollen season in 2008 (Birchseason) was included as 1 (April 15-May 15) and 0 (May 16-July 31), based upon birch pollen counts. In this analysis, 2007 was omitted since the birch pollen season of 2007 had ended when the collection of symptom scores started.

## Results

### Recruitment of patients

Relevant characteristics of the patients are listed in Table 1. In the skin prick test 19 (2007) and 18 (2008) percent of the patients responded exclusively to grass pollen, 65% to birch pollen, 55.5% to alder pollen, 12% to mugwort pollen and 30% to house dust mite. According to patient self-reports, oral antihistamines were the most frequently used medication (Table 1).

**Table 1:** Characteristics of the patient group

	2007	2008
Number	80	84
Mean age (SD)	33.5 (9.6)	34.5 (10.1)
Sex (M/F)	30/50	33/51
Mono grass pollen allergy^1^	19	18
Medication use^2^		
Oral antihistamine	63	65
Nasal antihistamine	4	3
Nasal cromoglicate	4	8
Nasal steroid	16	19
Eye drops antihistamine	5	9
Eye drops cromoglicate	8	9
Inhalation medication	5	3

^1^Number of patients exclusively positive in the skin prick test for grass pollen

^2^Number of patients indicating their specific medication use during the anamnesis

### Symptom scores

Patients sent in their daily symptoms scores either by SMS (21.7%) or by internet (78.3%). Although it was partly summer holiday season, the compliance of the patient group was high. During the 11 week-period in 2007 and the 15 week-period in 2008 the compliance was 78.8% and 77.3%.

**Figure 1:**
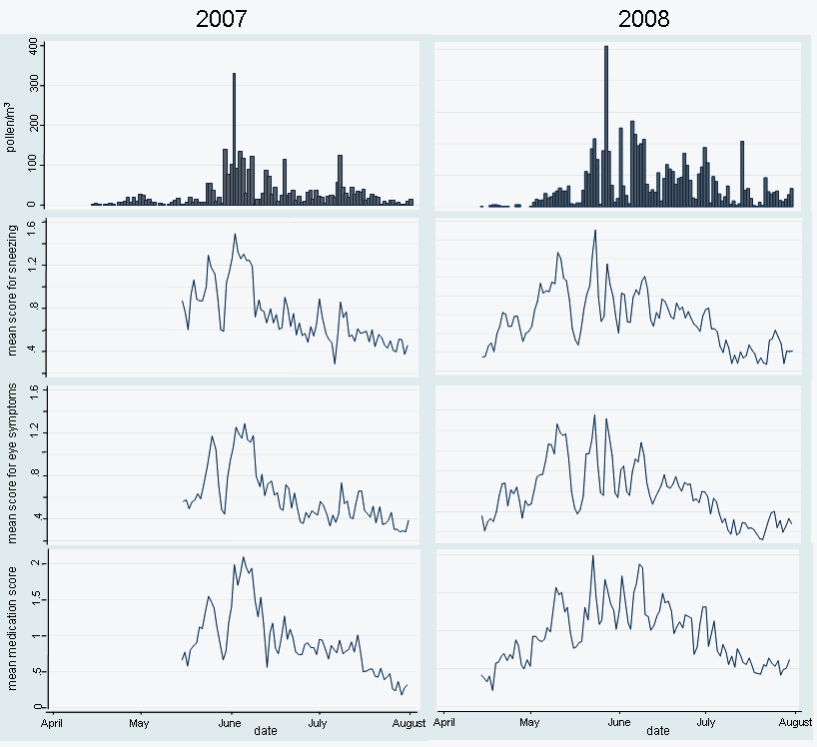
**Daily grass pollen counts and daily mean scores for sneezing, eye symptoms and medication use during the grass pollen season in 2007 and 2008**.

**Figure 2:**
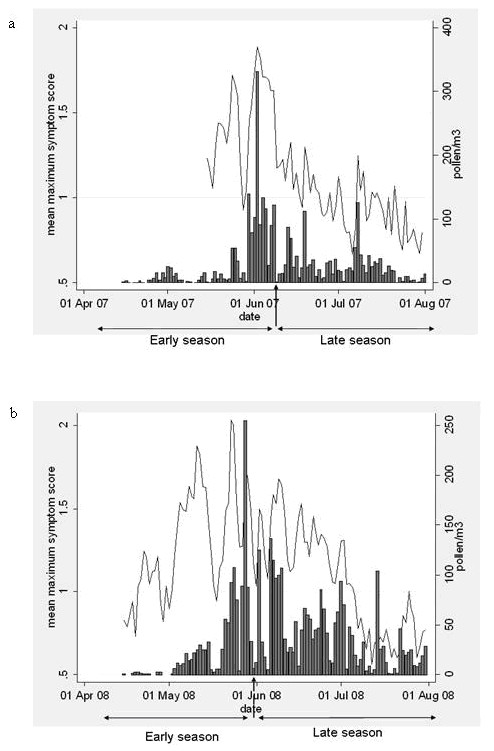
**Daily mean of the maximum symptom score (line) in 2007 (a) and 2008 (b)**. The bars show the daily grass pollen counts. The season is split into an early and late season on June 12^th ^in 2007 and May 31^st ^in 2008 (indicated by arrows).

The daily mean scores of each of the five symptoms during the season show a similar temporal pattern (e.g. eye symptoms and sneezing in Figure 1). In addition, the severity of the five symptoms correlate with the daily pollen counts, showing high symptom scores at high pollen concentration and low symptoms scores at low pollen concentrations (Figures 1, 2). Indeed, there was a significant relationship between daily pollen counts and the different symptom scores with the correlation coefficients ranging from 0.60 to 0.65 (p < 0.001) in 2007 and from 0.51 to 0.55 (p < 0.001) in 2008. Also, the daily mean medication use showed a similar temporal pattern, with higher medication use during days with high pollen counts and severe symptoms and reduced medication use on days with low pollen counts and milder symptom severity (Figure 1). The correlation coefficient between pollen counts and mean medication use was 0.57 (p < 0.001) in 2007 and 0.63 (p < 0.001) in 2008.

The mean maximum symptom score (range) was 1.13 (0.67-1.89) and 1.16 (0.56 - 2.03) in 2007 and 2008, respectively. The mean maximum symptoms score was significantly correlated with each of the five symptom scores with a correlation coefficient ranging from 0.86 for postnasal drip to 0.96 for sneezing. (p < 0.001) The correlation coefficient between the mean maximum symptom score and pollen count was 0.61 and 0.51 in 2007 and 2008, respectively, (p < 0.001).

**Figure 3:**
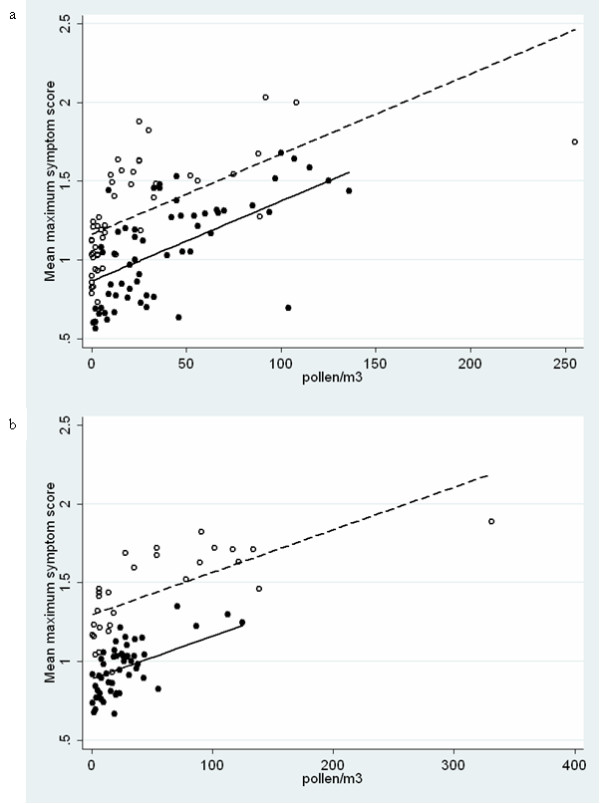
**Scatter plot of the daily mean of the maximum symptom score and the pollen concentrations in the early (open circles) and the late season (closed circles) of 2007 (a) and 2008 (b)**. Regression lines are shown for the early season (dashed line) and for the late season (solid line).

Figure 2 shows that in both years the symptoms scores at the beginning of the season in May after the first grass pollen exposure appeared to be higher than scores reported at similar grass pollen concentrations at the end of the season. This phenomenon was analysed in more detail. A multiple regression analysis with the mean maximum symptom score (dependent), the pollen (independent) and the period, i.e. the early and the late season (covariate), showed a significant period effect (p < 0.001). At similar grass pollen concentrations, symptoms were higher in the early season compared to the late season (difference: -0.41 [95% CI -0.42 to -0.40]; and -0.30 [95% CI -0.32 to -0.29] for 2007 and 2008, respectively). This is reflected by the observation that the relationship between the daily mean maximum symptom score and the daily pollen concentration is different in the two periods (Figure 3). The regression model explained 73% and 45% of the variance for 2007 and 2008, respectively.

**Figure 4:**
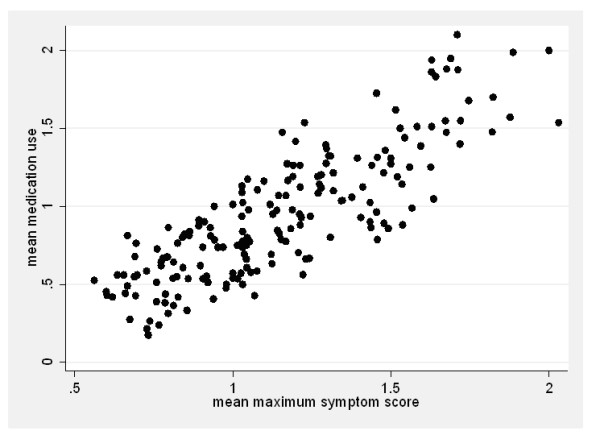
**The relationship between daily mean medication use and daily mean maximum symptom scores of the patient group in both the 2007- and 2008 pollen season (r = 0.84, p < 0.001)**.

Although medication use itself had an independent effect on maximum symptom scores (OR ≥ 2.77; p < 0.001), analyses with or without medication use revealed similar proportional odds ratios of the period effect (table 2). Therefore, medication use does not seem to be a confounder of the period effect. The mean maximum symptom scores showed a positive correlation with daily mean medication use (Figure 4), reflecting medication use (mainly antihistamines, table 1) upon development of symptoms. However, since frequent use of nasal steroids might have a long-term effect on symptoms, we assessed whether steroid use could explain the period effect. For both years, the proportional odds ratio of the period was not significantly affected by this potential confounder (table 2). The ordered logistic analysis in which patients using steroids were excluded showed similar results (proportional odds ratio for the period 0.43 [0.33-0.57] and 0.48 [0.37-0.61] for 2007 and 2008 respectively) as the analysis with the complete patient group (see table 2). These analyses show that neither the use of nasal steroids nor medication use in general explain the difference in symptom scores at similar grass pollen concentrations between the two periods of the season (table 2).

**Table 2:** Analysis of confounding factors related to medication use by ordered logistic regression with pollen and the period and one confounding factor at a time.

Confounding Factor	Factors of analysis	2007		2008	
		
		Proportional Odds Ratio (95%CI)	P-value	Proportional Odds Ratio (95%CI)	P-value
-	Period^a^	0.40 (0.32-0.50)	< 0.001	0.49 (0.40-0.61)	< 0.001

Medication	Period	0.42 (0.33-0.53)	< 0.001	0.48 (0.39-0.60)	< 0.001

	Medication use^b^	2.77 (1.74-4.43)	< 0.001	3.57 (2.47-5.17)	< 0.001

Nasal steroid	Period^a^	0.40 (0.31-0.51)	< 0.001	0.48 (0.38-0.59)	< 0.001

	Nasal steroid^c^	1.51 (0.76-2.97)	0.24	2.43 (1.26-4.70)	0.008

The proportional odds ratio, the 95% confidence interval (CI) and the p values for the period and the confounding factor for each model are shown for 2007 and 2008.

^a ^The period refers to the late (index) versus the early season (reference) of the grass pollen season.

^b^Medication use refers to the daily use of any medication for hay fever symptoms (index) versus no use of medication (reference).

^c^Nasal steroid refers to the daily use of nasal steroid spray (index) versus no use of nasal steroid spray (reference).

The grass pollen season is preceded by the birch pollen season. Since 65 percent of the patients were co-sensitized for birch pollen, a stratified analysis for birch co-sensitization was performed. This analysis did not show an effect of co-sensitization for birch pollen on maximum symptom scores in neither the 2007 nor the 2008 season (p > 0.1, table 3). Therefore, priming by birch pollen does not seem to be a confounder of the period effect. However, since the end of the birch pollen season and the start of the 2008 grass pollen season overlapped, we performed a stratified analysis of the group of patients co-sensitized to birch pollen and the overlapping birch and grass pollen period. This analysis showed a trend of an effect of birch pollen sensitization for this period on maximum symptom scores (p = 0.089). However, the proportional odds ratio of the period was not significantly affected (table 3). A stratified analysis of patients with mono grass pollen allergy did neither significantly influence maximum symptom scores nor did it explain the period effect (data not shown). Similar results were found in a stratified analysis with patients with a clinically relevant house dust mite allergy (see table 3).

**Table 3:** Analysis of effect modifiers related to the allergic sensitization of the patients by ordered logistic regression with pollen and the period and the effect modifier(s).

Effect modifier	Factors for analysis	2007		2008	
		
		Proportional Odds Ratio (95% CI)	P-value	Proportional Odds Ratio (95%CI)	P-value
-	Period^a^	0.40 (0.32-0.50)	< 0.001	0.49 (0.40-0.60)	< 0.001

Birch sensitization	Period^a^	0.32 (0.21-0.48)	< 0.001	0.53 (0.36-0.77)	< 0.001
	Birch^+b^	0.71 (0.31-1.59)	0.40	1.26 (0.64-2.48)	0.51
	Birch^+^_*_period	1.40 (0.83-2.38)	0.20	0.91 (0.56-1.44)	0.68

Birch sensitization and pollen season	Period^a^	ND^d^		0.52 (0.36-0.76)	< 0.001
	Birch^+ b^	ND		1.22 (0.61-2.43)	0.58
	Birchseason^c^	ND		0.92 (0.58-1.49)	0.76

Birch sensitization and pollen season	Period^a^	ND^d^		0.54 (0.43-0.68)	< 0.001
	Birch^+b^	ND		0.97 (0.59-1.58)	0.90
	Birchseason^c^	ND		0.89 (0.65-1.23)	0.49
	Birch^+^_*_Birchseason	ND		1.48 (0.94-2.33)	0.09

HDM	Period^a^	0.38 (0.29-0.50)	< 0.001	0.52 (0.41-0.66)	< 0.001
	HDM^e^	0.73 (0.27-1.93)	0.52	1.25 (0.55-2.84)	0.59
	HDM _* _period	1.27 (0.78-2.08)	0.34	0.68 (0.39-1.18)	0.17

The proportional odd ratio, the 95% confidence interval (CI) and the p values for the period and the effect modifier for each model are shown for 2007 and 2008.

^a ^The period refers to the late (index) versus the early season (reference) of the grass pollen season.

^b^Birch^+ ^refers to patients that are co-sensitized to birch pollen (index) versus patients with a negative skin prick test for birch pollen (reference).

^c^Birchseason refers to the birch pollen season (index) versus the period outside the birch pollen season (reference).

^d^ND = not determined since in 2007 the birch pollen season was over by the time the symptoms collection started.

^e^HDM refers to patients with a clinically relevant house dust mite allergy (index) versus patients without an house dust mite allergy (reference).

## Discussion

In the present study we found that grass pollen allergic patients have more severe symptoms in the early season compared to the late season at similar grass pollen concentrations. This seasonal effect could not be explained by any of the confounding factors including self-reported medication use, co-sensitization to birch pollen and a clinically relevant house dust mite allergy.

In this study, the between-day variations in five separate types of symptoms (runny nose, sneezing, blocked nose, postnasal drip, and eye symptoms) were summarized by the mean maximum symptom score. By selecting a patient's maximum symptom score, irrespective of the nature of the symptom, we were able to monitor the day-to-day variations in seasonal allergic rhinitis symptoms severity (see Figure 2). The late start of the symptom score collection in 2007 resulted in a lower number of observations in the early season compared to that period in 2008. A major drawback of this late start is the fact that the overlap in birch and grass pollen season could not be studied in this study year (see below). However, the results of 2007 and 2008 were consistent in showing more severe symptoms in the early season compared to the late season at similar grass pollen concentrations.

It was relevant to study whether the difference in symptom scores in the early and the late season of the grass pollen season that we observed could be due to confounding factors, such as the use of medication and co-sensitization to birch pollen or house dust mite in the patients. Although this latter could have been avoided by including primarily mono grass pollen sensitized patients, those patients appeared to be very rare. We also considered the possibility that a more regular medication use in the late period could reduce symptom severity. However, the ordered logistic regression analysis showed that medication use did not affect the period effect in the response to grass pollen. On the contrary, the patients using medication appeared to suffer from more severe symptoms than the patients who did not use medication. Furthermore, the self-reported medication use increased with increasing mean maximum symptom score (Figure 4), suggesting that the patients took their medication mainly on demand.

Birch co-sensitization appeared to significantly contribute to the maximum symptom score in 2008, when the birch pollen season overlapped with the start of the symptom collection and the grass pollen season. This result can be explained, because patients co-sensitized to birch pollen may respond in the overlapping season to the presence of birch pollen, while grass pollen concentrations are still low. However, the birch pollen sensitivity did not influence the period effect significantly (table 3). Therefore, we conclude that neither of these confounding factors could fully explain the differences in response to similar grass pollen concentrations between the early and the late season. Also selective loss of patients (e.g. mild patients in the early season) can be excluded as a cause for the observed difference in symptom scores between the early and late season, since the compliance among all patients in the group was very high (78.8% and 77.3% in 2007 and 2008, respectively).

The ICT based system by which the daily symptoms were recorded and transferred to the study centre at the end of each day by internet or by SMS, is a novel approach. In most studies symptom scores are collected using a weekly questionnaire or a diary [11]. This may lead to missing data and the data may contain a recall bias.

Very diverse explanations may account for the observed findings. The first could be the occurrence of a few days with very high grass pollen concentrations in the early season. Patients that experienced severe symptoms during this period may consider their symptoms on subsequent days in the late season much less severe. Secondly, during the season different grass species start to flower and produce pollen. Although the allergenic determinants of major allergens of group 1 and 5 are very similar in at least 12 grass pollen species studied^5^, the content of allergens (allergenic potency) of the grass pollen of different species may vary between those that produce pollen in the early versus the late season. The lower symptom score in the late season may be a reflection of the difference in the allergic response to the early and the late flowering species. Finally, according to the concept of priming, repeated exposure of the nasal tissue to allergen may cause a non-specific up-regulation of mucosal sensitivity and responsiveness [12,13] However, this priming effect is not reported consistently [14-18]. Most provocation studies do not extend more than 2 weeks, and the effect of repeated exposure beyond 2 weeks is not well known [12]. It has been suggested that especially grass pollen allergic patients may have a natural potential to down regulate their allergic response after repeated allergen exposure [14]. The high exposure to grass pollen in the end of the early season may result in a decrease in allergic inflammation during subsequent exposures in the late season. Whether mechanisms similar to those observed after immunotherapy, i.e. an increase in blocking antibodies and regulatory T cells [19,20], are implicated in this decreased inflammation is unknown. However, the observation of an increase in Foxp3+ T regulatory cells in the nasal mucosa of patients during the grass pollen season compared to patients outside the season [21] indeed suggests that allergen-specific regulatory T cells reduce the allergic response during the late season.

## Conclusion

We found strong evidence that grass pollen allergic patients have more severe symptoms in the early season compared to the late season at similar grass pollen concentrations. This finding has implications for our understanding of symptom development and for clinical studies during the grass pollen season. A difference in response to grass pollen during the study period may interfere with the outcome of such clinical studies. Careful planning of such studies is crucial for adequate interpretation of the results. Furthermore our findings have implications for the development of forecasts for seasonal allergic rhinitis symptoms. The different response to grass pollen during the grass pollen season should be taken into account when the forecast of seasonal allergic rhinitis symptoms is based on expected grass pollen counts.

## Competing interests

The authors declare that they have no competing interests.

## Authors' contributions

LAdW wrote the first version of the manuscript, LAdW, IT, PSH and JKS contributed to the design of the study, interpretation of the data, preparation and critical revision of the manuscript. LAdW, IT, TB, JMG-S and DTvdP were involved in collection of the data, LAdW, TB and JKS analysed and interpreted the data. All authors approved the final version of the manuscript.
